# Supplementary material for: Isolation and Pathogenicity Analysis of a G5P[23] Porcine Rotavirus Strain
Source: Viruses. 2023 Dec 22;16(1):21. doi: 10.3390/v16010021 (PMC10819142; doi:10.3390/v16010021)
Supplement: Supplementary file 1 [file viruses-16-00021-s001.zip › viruses-2724439-supplementary.pdf]

Table S1. Sequences of primers and probes used in this study.

Primer Name	5′- sequence –3′	Target	Reference
PDCoV-RTF	5′-CGACCACATGGCTCCAATTC-3′		
PDCoV-RTR	5′-CAGCTCTTGCCCATGTAGCTT-3′	M	[1]
PDCoV-RTP	5′-FAM-CACACCAGTCGTTAAGCATGGCAAGC-BHQ-1-3′		
PEDV-RTF	5′-GATACTTTGGCCTCTTGTGT-3′		
PEDV-RTR	5′-CACAACCGAATGCTATTGACG-3′	M	
PEDV-RTP	5′-FAM-TTCAGCATCCTTATGGCTTGCATC-BHQ1-3′		[2]
TGEV-RTF	5′-TGCCATGAACAAACCAAC-3′		
TGEV-RTR	5′-GGCACTTTACCATCGAAT-3′	N	
TGEV-RTP	5′-FAM-FATAGCACCACGACTACCAAGC-BHQ1-3′		
RVA-RTF	5′-GCTAGGGAYAAA ATTGTTGAAGGTA-3′		
RVA-RTR	5′-ATTGGCAAATTT CCTATT CCTCC-3′		[3]
RVA-RTP	5′-FAM-ATGAATGGA AATAATTTTCAAAC-BHQ1-3′	VP6	
RVA-RTF	5′-GGCTTTTAAACGAAGTCTTC-3′		This study
RVA-RTR	5′-GGTCACATCCTCTCACT-3′		

Table S2. Comparison of nucleotide and amino acid characteristics of RVA/Pig/China/JS/2023/G5P[23] strain with the closest strains in the GenBank database**.**

Gene	Genotype	Closely Related Strains	Nucleotide (%)	Amino acid (%)	Accession
VP1	R1	RVA/Pig-tc/USA/Gottfried/1983/G4P6	99.0	94.8	KR052746
VP2	C1	RVA/Pig-tc/USA/OSU/1977/G5P7	98.4	92.1	KR052758
VP3	M1	RVA/Human-tc/USA/Wa/1974/G1P8	96.8	87.4	MT796880
VP4	P[23]	P23-RVA/Pig-tc/VEN/A34/1985/G5P23	84.5	89.5	AY174094
VP6	I5	RVA/Pig-tc/MEX/YM/1983/G11P7	90.5	89.1	X69487
VP7	G5	RVA/Human-tc/BRA/IAL28/1992/G5P8	91.9	87.2	EF672588
NSP1	A8	RVA/Pig-tc/MEX/YM/1983/G11P7	93.2	89.3	D38154
NSP2	N1	RVA/Human-wt/BGD/Dhaka25/2002/G 12P8	90.1	95.5	DQ146656
NSP3	T1	RVA/Pig-tc/MEX/YM/1983/G11P7	87.6	88.9	GU199518
NSP4	E1	RVA/Human-tc/USA/P/1974/G3[P8]	89.8	88.1	EF672603.1
NSP5	H1	RVA/Pig-tc/USA/Gottfried/1983/G4P6	89.5	96.1	KR052745

References:

1. Zhu, J.H.; Rawal, G.; Aljets, E.; Yim-Im, W.; Yang, Y.L.; Huang, Y.W.; Krueger, K.; Gauger, P.; Main, R.; Zhang, J.Q. Development and Clinical Applications of a 5-Plex Real-Time RT-PCR for Swine Enteric Coronaviruses. *Viruses* **2022**, *14*, 1536. https://doi.org/10.3390/v14071536.
2. Huang, X.; Chen, J.N.; Yao, G.; Guo, Q.Y.; Wang, J.Q.; Liu, G.L. A TaqMan-probe-based multiplex real-time RT-qPCR for simultaneous detection of porcine enteric coronaviruses. *Appl. Microbiol. Biotechnol.* **2019**, *103*, 4943–4952. https://doi.org/10.1007/s00253-019-09835-7.
3. Marthaler, D.; Homwong, N.; Rossow, K.; Culhane, M.; Goyal, S.; Collins, J.; Matthijnssens, J.; Ciarlet, M. Rapid detection and high occurrence of porcine rotavirus A, B, and C by RT-qPCR in diagnostic samples. *J. Virol. Methods* **2014**, *209*, 30–34. https://doi.org/10.1016/j.jviromet.2014.08.018.
